# The Bedding and Linen Department

**Published:** 1908-05-02

**Authors:** 


### Nursing Administration.

### THE BEDDING AND LINEN DEPARTMENT.

### I.—MUDDLE OR ORGANISATION.

This is one of the most arduous branches of the Matron's domain. It is also the department concerning which in all probability her training has afforded her least experience. Many a matron passes from hospitals where all matters relating to the linen department have been so well organised that the organisation is not perceptible, to take up duties in an institution where no system worthy of the name prevails, and has found herself compelled to educe order out of chaos by the process of thinking it all out for herself and buying experience at the dear price of constant blunders. The very composition of the beds is often unknown to the sister whose principal duties centre round them. And when, in some new post, she finds the responsibilty thrown upon her of bringing the bedding and linen into good order, she is confronted with a sense of her colossal ignorance.

The first step undoubtedly to a perfect linen department is to form a clear idea of quantities. To have enough for all possible contingencies, and yet not to cumber the limited store-room accommodation with a surplus is a serious problem, and it is one which cannot be settled off hand, since the size of the hospital, its situation, and its distinctive character, together with the class of patients received, all modify the required supplies. Hospitals in large smoky towns need larger supplies than those in the country or quiet Cathedral cities. The clinical hospital makes larger demands on the linen cupboard than the non-clinical. The number of acute surgical cases received is a factor not to be ignored, and the larger the institution, according to some curious law, the more liberal the proportion of necessaries per bed must be. Having compiled an inventory of every article which can conceivably be required by any patient, doctor, nurse, or maid, in any department of the institution, the matron finds herself confronted with the responsibility of choosing for each article amid a bewildering variety of samples the exact material which represents the minimum of cost combined with the maximum of wear. To be parsimonious in this selection is, she is well aware, the worst possible economy. Yet she must distinguish between the higher price which only means extra "finish," and that which may mean an extra year or two of wear.

This needs an expert eye, and is one of the things which is done better in France, where the hospitals are served from a central store managed in the interests of the institutions, and prices are kept as low as a guaranteed standard of high quality will admit. In small hospitals where the matron is a practical housekeeper and is able to devote her attention to these details, the best results attainable are often to be found. But where samples are pronounced on by persons who have no experience in their use, the amount of money thrown away by bad buying is beyond computation. When quantity and quality have both been settled the matron often encounters a serious obstacle in reorganising the linen department owing to the reluctance of the Board to vote her sufficient money to place it on a really satisfactory basis. It cannot be denied that institutions can muddle on under a shortage of every necessary, and yet accomplish good work. When this has continued for a term of many years the matron who desires to reform matters will be regarded as recklessly extravagant in her estimate of the sum needed to place things on a good footing. She must either wait and contrive as her predecessors have done, or devise some means of supplying the linen without resort to the general funds. This has been most successfully achieved in many places by the aid of the Ladies' League.

Let it be granted that the linen department is in thorough order. How long will it remain so? The linen in a hospital is exposed to great trials. It is torn, injured by chemicals, lost or stolen, it deteriorates from sheer wear and tear. The matron must be perpetually on the watch for articles which need mending, to check the supplies for each ward periodically, and to make good deficiencies. The secret of the proper control of the linen department depends on the provision of proper accommodation, and when the vicissitudes of a sheet be considered in the week's routine it will be seen that this is a big question. For the sheet must pass from some central store-room to the shelf where it will be available for use by the sister of the ward. Thence it is placed on the patient's bed. Thence it passes to some receptacle for the soiled linen of the ward, or, should the case have been an infectious one, to a spot where it can be immersed in disinfectant. Again it will move on to the soiled-linen room, where it will be counted and despatched to the laundry, and on returning from the laundry it will find an eighth resting-place before it has been checked among its fellows, and finds itself once more back again in the ward-linen cupboard. At every one of these stages something adverse may occur.

The matron, who has a good grip over the department, will keep in view the extravagance of some sisters in the use of linen, and will compare the amount used in one ward with that used in another. She will be particular about the condition in which the linen returns from the laundry, and will not tolerate that muddy and creased appearance often seen in linen placed fresh upon the beds. Who after this brief enumeration of its difficulties will maintain that the linen department is only a minor branch of the hospital economy? In large institutions the entire time of an able sister will be needed to cope with its intricacies, and even in small establishments the matron ought to be able to command some skilled assistance in regulating it, if the results are to be entirely satisfactory.

